# Investigation of Cytotoxicity of Phosphoryl Choline Modified Single-Walled Carbon Nanotubes under a Live Cell Station

**DOI:** 10.1155/2014/537091

**Published:** 2014-06-25

**Authors:** Yufeng Zhao, Qunlong Mao, Yu Liu, Yan Zhang, Tao Zhang, Zhengsheng Jiang

**Affiliations:** ^1^College of Engineering and Applied Sciences, Nanjing University, Nanjing 210093, China; ^2^Nanjing Excellence Technology Center for Interventional Medical Devices, Nanjing 210093, China; ^3^Physics Department, Nanjing University, Nanjing 210093, China

## Abstract

Single-walled carbon nanotubes (SWCNTs) and various modified SWCNTs have drawn a lot of attention due to their potential applications in biomedical field. Before further moving on to real clinical applications, hydrophobicity and toxicity of SWCNTs should be investigated thoroughly. In this paper, 2-methacryloyloxy ethyl phosphorylcholine (MPC) was adopted to modify SWCNTs and phosphoryl choline was grafted onto SWCNTs as small molecule moieties and polymeric chains, which made SWCNTs dispersed stably both in water and in cell culture medium for a long time. Cytotoxicity of pristine and modified SWCNTs were assayed upon successful preparation of the designed modified SWCNT. Furthermore, the internalization of SWCNTs by three cells was investigated using a live cell station under normal culture temperature (37°C) and low temperature (4°C). The results showed that the internalization of modified SWCNTs was related to both the active transport and the passive transport. Although the modification with phosphoryl choline remarkably reduced the cytotoxicity of SWCNTs, the results were probably due to other reasons such as the decrease in the ratio of cells which internalized modified SWCNTs since the cells without SWCNTs occupation still exhibited normal states.

## 1. Introduction

Since the discovery of carbon nanotubes (CNTs) by Iijima in 1991 [1], both multiwalled CNTs and single-walled CNTs (SWCNTs) have drawn a lot of attention in different areas, among which biomedical engineering is one of the most prominent CNTs [2–7]. However, CNTs are not soluble or even dispersible in water due to their inclination of aggregation which resulted from their hydrophobicity. This phenomenon is especially troublesome to SWNTs by severely impeding their potential improvements for biomedical devices. Moreover, the concerns on the toxicity of nanomaterials including CNTs [8–12] are another problem that needs to be solved before real clinical application of CNTs as biosensor, drug delivery, and biomedicines. In order to deal with these problems, various physical and chemical modifications [13] on CNTs have been carried out for the purpose of improving their solubility in water, which will in turn partially resolve toxicity issue. For example, hydroxyl group [14], carboxyl group [15], polyethylene glycol [16–22], amino group [23], poly(allylamine) [24], DNA or RNA [25, 26], and many others [13, 27] have been used to functionalize CNTs. Meanwhile, the studies on the toxicities, bioeffects, and environment health risks about CNTs have also made great progresses in the past few years; the studies themselves have moved from* in vitro* model and statistical assay to* in vivo* animal studies and mechanisms investigations [11, 28–31].

Many researchers have been focusing on the cytotoxicity of CNTs and quite a few papers have been published, but controversies have been seen in their studies. For example, some studies showed that CNTs decrease cell viability [9, 32–34] and upregulate genes associated with inflammation and apoptosis [34–37], while others have shown minimal or no decrease in cell viability [38, 39]. As a result, it is exceptionally difficult to evaluate CNTs toxicity based on current existing data. To make the case even more complicated, the designs of toxicity studies, such as the physicochemical characterizations [40], cytotoxicity assays [41], and the definition of toxicity itself [42], have aroused controversies. Unfortunately, despite the complexity and expense of evaluating toxicity of various CNTs modifications, it is essential to establish a general relationship between cytotoxicity and physicochemical properties of nanotubes as a method to lower the risk of commercializing CNTs biomedical devices. The establishment of cytotoxicity-physicochemical property relationship requires simultaneous material characterization and standardized toxicity evaluations. Deliberately documented and well-defined studies will also induce helpful mechanistic understanding of nanomaterial toxicity [43].

In our previous studies [44–46], phosphoryl choline moieties were grafted onto MWCNTs and then cytotoxicity of the modified CNTs was evaluated in cell and mice. It was found that the functionalization on MWCNTs with phosphoryl choline significantly reduced the toxicity of CNTs. Our results indicated that phosphoryl choline moiety was a valuable modifier for CNTs regarding their biomedical applications. In this paper, the similar modification was applied on SWCNTs; not only the basic cytotoxicity evaluation, but also the cell internalization mechanism was discussed.

## 2. Experimental

### 2.1. Materials

SWCNTs were purchased from Shenzhen Nano-Technologies Port Co., Ltd. (Shenzhen, China) which were prepared via chemical vapor deposition (CVD) with La–Ni catalyst and the purity is no less than 97% with about 3% amorphous carbon. 2-Methacryloyloxy ethyl phosphorylcholine (MPC) without inhibitor was supplied by Joy-Nature Institute of Technology (Nanjing, China) with purity at 96% and used as received. The other chemicals, including sulfuric acid (H_2_SO_4_), nitric acid (HNO_3_), thionyl chloride (SOCl_2_), ethanolamine (HOCH_2_CH_2_NH_2_), ethanediamine (NH_2_CH_2_CH_2_NH_2_), 2-bromoisobutyryl bromide ((CH_3_)_2_CBrCOBr), triethylamine (TEA, (C_2_H_5_)_3_N), tetrahydrofuran (THF), cuprous bromide (CuBr), and dimethylsulfoxide (DMSO), were purchased from Sinopharm Chemical Reagent Co. (Shanghai, China) and purified according to references [47] if necessary. Fluorescein isothiocyanate (FITC) was purchased from Aladdin Industrial Corporation (Shanghai, China).

Human umbilical vein endothelial cells (HUVEC) were obtained from Lifeline Cell Technology Co. (distributed by Beijing Qingyuanhao Biologics, Beijing, China). While cell lines of murine macrophage RAW264.7 and murine fibroblast L929 were obtained from the ATCC (distributed by Beijing Zhongyuan Limited, Beijing, China), Dulbcco's Modified Eagle's medium (DMEM, with 4500 mg/L-glucose and L-glutamine) and VEGF LS-1020 culture medium were purchased from Thermo Fisher Scientific China Branch (Shanghai, China). Fetal bovine serum (FBS) was purchased from Hangzhou Sijiqing Biological Engineering Materials Co., Ltd. (Hangzhou, China), which was heat inactivated for 30 min at 56°C and then stored at −20°C before use. 0.25% trypsin-EDTA (1X) with phenol red, penicillin/streptomycin (100X), was purchased from Life Technologies Co., Shanghai Branch (Shanghai, China). Cell Counting Kit-8 (CCK-8) was purchased from Dojindo Molecular Technologies, Inc. (Shanghai, China).

### 2.2. Instruments and Methods

The X-ray photoelectron spectra (XPS) of SWCNT-PC were recorded on a VG Scientific ESCALab MK-II spectrometer (West Sussex, England) equipped with a monochromatic Mg-K*α* X-ray source. Raman spectra were recorded on a JY HR800 laser Raman spectroscopy (Jobin Yvon Ltd., France). Thermogravimetric analysis (TGA) was done with a PE Pyris 1 thermogravimetor (PerkinElmer Life and Analytical Sciences, Inc., MA, USA) in a N_2_ atmosphere at heating a rate of 20°C/min from room temperature to 600°C. The content of metal and other impurity elements were recorded on ARL-9800 X-ray fluorescence spectrometer (ARL Ltd. Switzerland). The micromorphology images were taken on a JEM-2100 (JEOL Ltd., Tokyo, Japan) transmission electron microscope (TEM). The cells used in cytotoxicity evaluations were cultivated in a CO_2_ incubator (Heracell model 150i; Thermo Scientific, MA, USA) and the evaluations were carried out with a RT-6000 microplate reader (Rayto Ltd., Shenzhen, China) at a wavelength of 450 nm. The photographs were taken on an Axio Scope A1 microscope (Zeiss Ltd. Germany) with a water immersion/dipping lens (Zeiss W Plan-Apochromat 40x/1.0 DIC VIS-IR objective).

### 2.3. Preparation of Samples

**Scheme 1:**
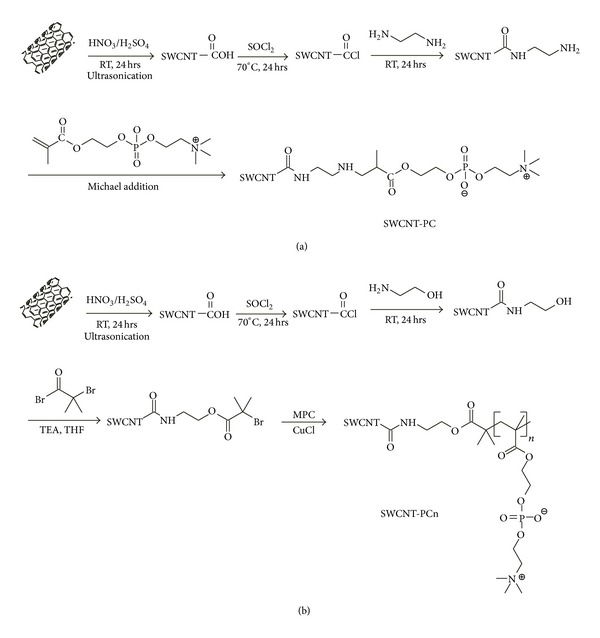
Synthesis routes of SWCNT-PC and SWCNT-PCn.

Two kinds of phosphoryl choline moieties modified SWCNTs were prepared according to the synthesis routes shown in Scheme 1. SWCNT-PC was synthesized following the steps including carboxylation, chloroacetylation, and amidation of SWCNTs; the brief reaction condition was presented in Scheme 1(a) and the details were described in reference [45]. For the step of Michael addition of MPC, ethanediamine grafted SWCNTs (100 mg) were reacted with MPC (1 g) in anhydrous ethanol (100 mL) for 48 hours at room temperature and then precipitated, centrifuged, and washed with acetone for three times to eliminate the unreacted reagents. In the final products, the phosphoryl choline moiety was grafted onto SWCNTs as a small molecule structure. As for SWCNT-PCn, the phosphoryl choline moiety was grafted onto SWCNTs as a polymeric chain on the grafting point of SWCNTs following the atom transfer radical polymerization (ATRP) of MPC as described by Narain et al. [48] and Zhu et al. [49] and briefly presented in Scheme 1(b).

In order to investigate the cell uptake of modified SWCNTs, FITC was connected to the modified SWCNTs utilizing the residual second amine groups. In the label process, SWCNT-PCn was dispersed in deionized water at concentration of 20 mg/mL and the dispersion was modulated to pH 9; freshly prepared FITC/DMSO (1 mg/mL) was added and kept stirring for 24 hours in dark at 4°C. The reactants were dialyzed with deionized water for a week and then vacuum dried and marked as SWCNT-PCn-FITC.

### 2.4. Cell Cultivation

RAW264.7 and L929 cells were inoculated into DMEM culture medium containing 10% FBS and 1% penicillin/streptomycin in 25 cm^2^ cell culture flasks and incubated at 37°C with 5% CO_2_. In the culture of HUVEC, the cells were suspended in VEGF LS-1020 culture medium. When the cells adhered to and overspread on the plate, the culture medium was removed and 0.25% trypsin-EDTA solution was added. After 2-3 min the digestion was terminated by adding DMEM culture solution containing 10% FBS; the solution was bubbled to form a single-cell suspension and then diluted and counted. The viable cells were planted at a density of 5 × 10^3^/mL and cultivated for 24 hours before the cells in exponential phase of growth and were exposed to pristine SWCNTs, SWCNT-PC, and SWCNT-PCn.

### 2.5. Assessment of Cytotoxicity

Pristine SWCNTs, SWCNT-PC, and SWCNT-PCn were dispersed in water by ultrasonication at a concentration of 10 mg/mL. To overcome the poor dispersibility of SWCNTs in water, the surfactant SDS was added at a concentration of 0.1 mg/mL, while no surfactant was added to the SWCNT-PC and SWCNT-PCn dispersion. Then five dilutions, 5, 10, 20, 40 and 80 *μ*g/mL, of the pristine SWCNTs, SWCNT-PC, and SWCNT-PCn were vortex-mixed, respectively, and each sample was added to each well of multiwell plates (200 *μ*L/well in 96 multi-well plates) and cultured for 24 h at 37°C. The supernatants were removed, and 0.01 mol/L PBS was used to clean the SWCNT residue. Then 20 *μ*L of 5 mg/mL CCK-8 in culture medium was added to each well and incubated for another 1 h at 37°C. The absorbance of the solution at 450 nm was recorded with a Rayto RT-6000 microplate reader. Every experiment was performed in sextuplicate.

### 2.6. Fluorescent Microscopy Investigation

The viable cells were planted at density of 5 × 10^3^/mL in 6 cm cell culture dishes (5 mL/dish) and cultivated for 5 h; then 10 *μ*L SWCNT-PCn-FITC dispersions (10 *μ*g/mL) were added to each dish and continued being cultured at 37°C or 4°C. At 4th hour and 20th hour, the cells were investigated under microscopy with a live cell station which could control the temperature and CO_2_ concentration, and the photographs were taken with a water immersion/dipping objective lens.

## 3. Results and Discussion

### 3.1. Sample Preparation and Characterizations

The samples used in this study were firstly characterized for their size, purity, Zeta potential, and metal impurities. The results were summarized in Table 1.

#### 3.1.1. XPS Analysis of SWCNTs and Modified SWCNTs

**Figure 1:**
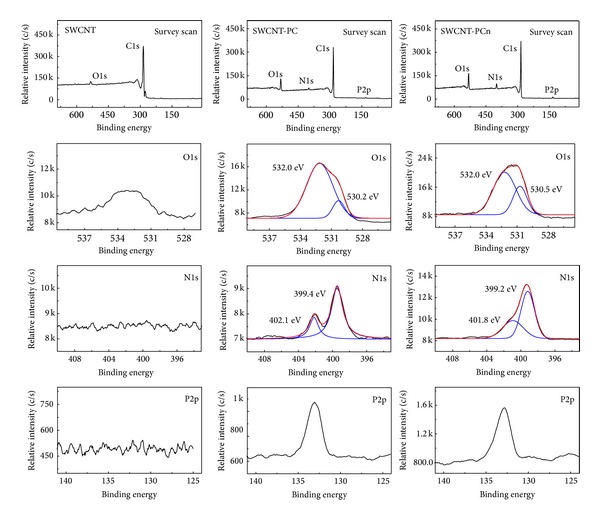
XPS spectra of SWCNT, SWCNT-PC, and SWCNT-PCn.

The XPS of pristine SWCNTs, SWCNT-PC, and SWCNT-PCn was recorded to confirm the linkage between different elements.  Figure 1 shows the survey scans and the region scans of SWCNT, SWCNT-PC, and SWCNT-PCn which confirms the existence of carbon, oxygen, nitrogen, and phosphorus elements in modified SWCNTs. But for pristine SWCNTs, only carbon and very seldom oxygen due to the structure defect could be observed. It indicated the successful introduction of phosphoryl choline moieties in SWCNT-PC and SWCNT-PCn samples. For both phosphoryl choline moieties modified samples, the O1s XPS spectrum showed a peak at the binding energy of 532.0 eV that belonged to the single bonds of C–O and/or P–O bonds and the humps at the binding energy of 530 eV which was the contribution of double bonds of C=O and/or P=O bonds [50]. Moreover, the higher ratio of double bonds to single bonds in SWCNT-PCn demonstrated that there are more phosphoryl choline structures, which in turn confirmed the existence of polymerized PC chain structure. Similar to O1s spectrum, the binding energy hump at 402 eV on the N1s XPS spectrum corresponded to N–H bonds in SWCNT-PC and SWCNT-PCn, while the binding energy peak at 399 eV corresponded to C–N bonds. A distinct peak for phosphorus 2p at 132.5 eV confirms the presence of the phosphorus containing bonds in both samples. The higher peak in SWCNT-PCn also proved the repeated structure of PC moieties.

#### 3.1.2. Raman Spectrum Inspection

**Figure 2:**
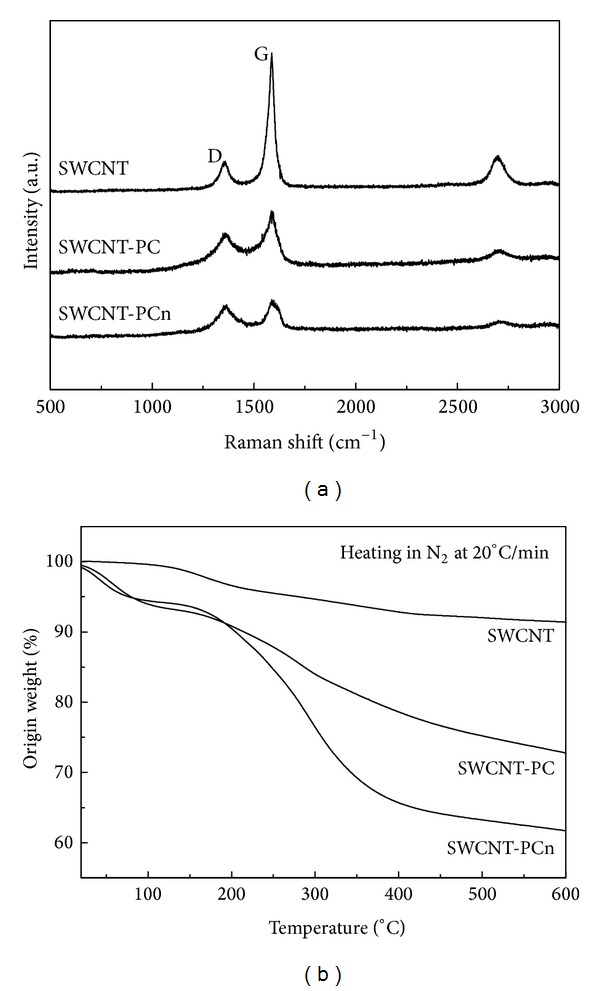
Raman spectra (a) and TGA curves (b) of SWCNT, SWCNT-PC, and SWCNT-PCn.

The existence of covalent functionalization can be inspected by Raman spectra [17]. As shown in Figure 2(a), the Raman spectrum of the SWCNTs exhibits a tangential mode (G band) at 1590 cm^−1^ and a relative weak disorder mode at 1300 cm^−1^ (D band) that was probably caused by defects formed during either the synthesis or the purification of the nanotubes. After modification, the relative intensity of the G band at 1590 cm^−1^ decreased, corresponding to the covalent attachment of the functional groups. If D/G ratio was defined as the ratio of the integrated peak area of D bands divided by the integrated peak area of G bands, it could be found that the larger D/G ratio for SWCNT-PCn and SWCNT-PC (from 3.38 of SWCNT-PCn to 1.07 of SWCNT-PC and to 0.78 of SWCNT) implies that the reaction with phosphoryl choline moieties leads to a higher level of functionalization.

#### 3.1.3. Thermogravimetric Analysis

The TGA curves of raw SWCNTs and modified SWCNTs were recorded and shown in Figure 2(b). In the total heating process, the weight of pristine SWCNTs lost about 9% owning to the amorphous carbon residue. For the curves of modified SWCNTs, the hydrophilicity of samples increased after the grafting process. The weight losses of SWCNT-PC and SWCNT-PCn before 150°C were about 7.8% and 7.6%, respectively, which was mainly due to the saturated water absorption. At higher temperatures, the weight loss should be due to the thermal decomposition of the side groups. Up to 600°C, the value was 28% and 38%. It may be concluded that weight loss increases with increased amount and size of the side groups grafted onto SWCNTs.

### 3.2. Dispersibility and Micromorphology of SWCNTs, SWCNT-PC, and SWCNT-PCn

**Figure 3:**
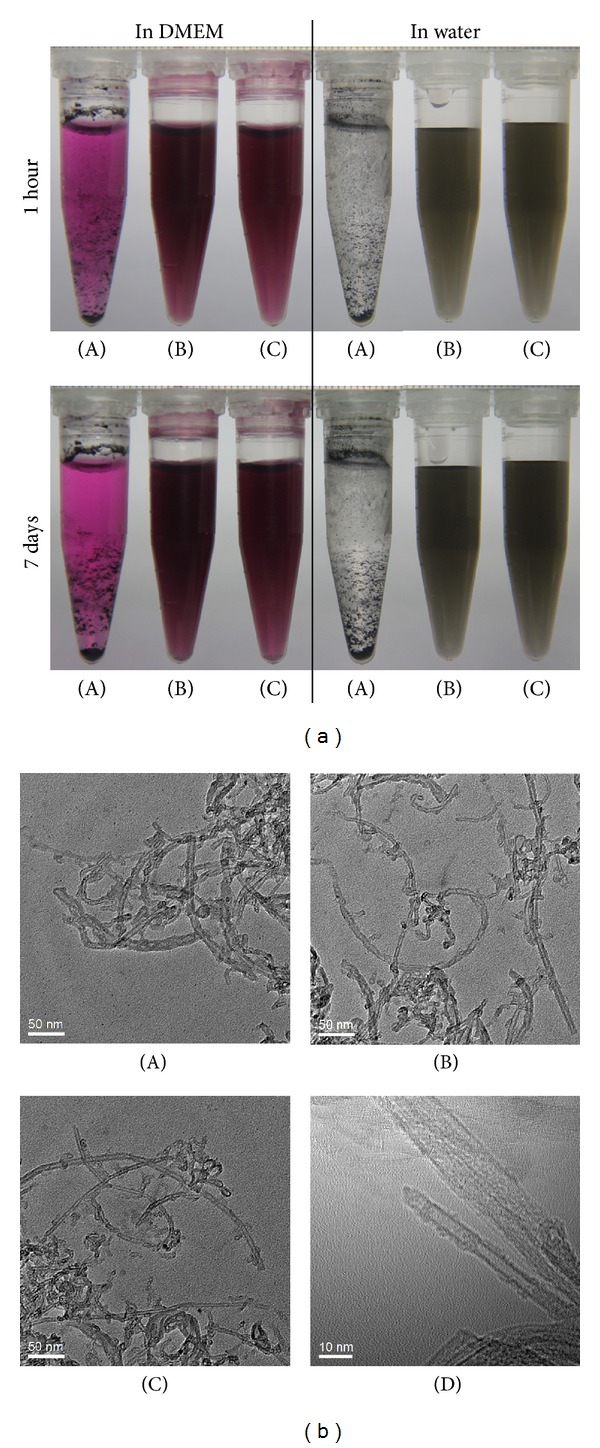
Dispersibility (a) of SWCNTs (A), SWCNT-PC (B), and SWCNT-PCn (C) in cell culture medium (DMEM) and water. (b), the TEM images of SWCNT (A), SWCNT-PC (B), SWCNT-PCn (C), and magnified SWCNT-PCn (D).

After modification with phosphoryl choline moieties, the insoluble pristine SWCNTs could easily disperse homogenously in water and DMEM, a cell culture medium. As shown in Figure 3(a), the pristine SWCNTs at concentrations of 5 mg/mL aggregated on standing 1 h after being dispersed by ultrasonication. However, dispersions of SWCNT-PC and SWCNT-PCn at the same concentrations were stable 7 days after being dispersed. In fact, the samples were still stable even two months later with just a sign of precipitation. Additionally, we also investigated the dispersion under centrifugation. It was found that SWCNT aggregated soon after ultrasonication and precipitated completely when centrifuged at 2500 ×g for 5 minutes. However, dispersion of SWCNT-PC and SWCNT-PCn at concentration of 5 mg/mL was stable after centrifugation and when left at least 10 days.

The stable dispersion of SWCNT-PC and SWCNT-PCn can be attributed to the hydrophilic properties of the phosphoryl choline moieties. The side groups extend into the solution because of the strong solvation of water on the PC moiety making the modified SWCNTs dispersion stable.

Actually, modification of SWCNTs with grafting water-soluble substance is one of the most effective methods among many ways to help SWCNTs dispersing in water. The reason for our choice of phosphoryl choline moieties as the modifier to accomplish the mission is phosphoryl choline's well-known biocompatible and zwitterionic properties. Zhao et al. [51] reported the synthesis of water-soluble SWCNTs with zwitterionic poly(*m*-aminobenzene sulfonic acid) graft copolymer and then found that its water solubility is about 5 mg/mL [18]. Similarly zwitterionic group, phosphoryl choline moieties, provides SWCNTs with similar solubility. Moreover, the biocompatibility of PC moiety is expected to improve the biocompatibility of SWCNT itself; thus by using three different kinds of cell lines, the cytotoxicity of SWCNTs and modified SWCNTs were investigated.

Figure 3(b) shows the TEM images of SWCNTs and modified SWCNTs. The dispersibility and morphology of modified SWCNTs are similar but the pristine SWCNTs are slightly entangled. Although many recent studies show that SWCNTs toxicity is highly associated with morphological signatures, we do not think morphology in this experiment which had a major impact on toxicological effects.

### 3.3. Cytotoxicity of SWCNTs and Modified SWCNTs: CCK-8 Assay

Three different cell lines, HUVEC, RAW264.7, and L929 cells, were chosen as our model cell lines to evaluate the cytotoxicity of SWCNTs as well as modified SWCNTs. In the* in vivo* toxicity evaluation of a hazardous materials, intravenous injection method is one of the most commonly used methods. In this method, endothelial cells were the first to contact the hazardous materials except for the blood components such as proteins, platelets, and red cells; therefore HUVEC was chosen by us to investigate the cytotoxicity for the purpose of evaluating their response to SWCNTs and modified SWCNTs. RAW264.7 and L929 are widely used in studies of cytotoxicity, endocytosis, metabolism, proliferation, and RNA expressions; we chose them to provide comparable results with the previous studies.

The cytotoxicity induced by pristine SWCNTs and the modified SWCNTs were determined by the CCK-8 assay, which is based on mitochondrial dehydrogenases activity. For a living cell, the succinate dehydrogenase in mitochondria may reduce exogenous CCK-8 and form water soluble orange formazan, while a dead cell, by definition, does not have such activity. The quantity of formazan formed is directly proportional to the quantity of living cells, which can be determined by directly measuring the absorbance at 450 nm.

**Figure 4:**
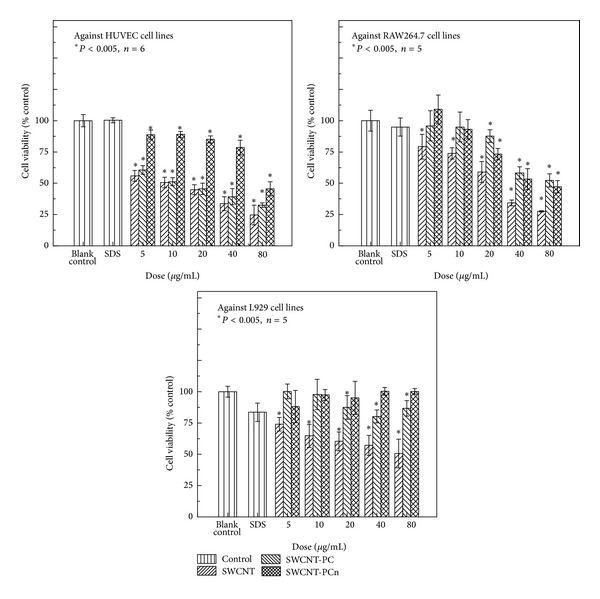
Cell viability after incubation of HUVEC, RAW264.7, and L929 cell lines with SWCNTs, SWCNT-PC, and SWCNT-PCn in variable dose for 24 h.

Figure 4 shows the results of the CCK-8 assay, the amounts of living cells exposed to SWCNTs, and modified SWCNTs for 24 h which were normalized according to the blank control group and the results were statistically analyzed with Student's* t*-test method. Due to the poor dispersion properties of SWCTS, surfactant, sodium dodecyl sulfate (SDS) at the concentration of 0.1 mg/mL was used to disperse the pristine SWCNTs, and no obvious cytotoxicity was found in the SDS group in all three cell lines.

Upon addition of pristine SWCNTs, the cell livability of three cell lines decreased remarkably and kept decreasing as the dosages of pristine SWCNTs increased. This observation indicated that pristine SWCNTs do shows dose-dependent cytotoxicity on the three cell lines tested. However, when SWCNTs were modified with grafting phosphoryl choline moieties, either PC or PCn cytotoxicity reduced: the cell livability of three cell lines increased compared to pristine SWCNTs at the same dosage. Moreover, the PCn moieties with chain structure attached to SWCNT showed much stronger effects on reducing the cytotoxicity than that of PC moieties with small molecule structure. Two possible explanations for these observations were made. The first possibility is that as more repeating phosphoryl choline units were introduced, the proportion of the hydrophilic groups in the modified moieties increased, which in turn led to higher hydrophilization of the SWCNTs' surface and weakened the interactions between carbon nanotube and cells. The other explanation is that, for the SWCNT-PCn sample, the hydrophobic polyacrylate carbon chains of PCn moieties tend to wrap the surface of SWCNT while the hydrophilic phosphoryl choline groups tend to detach the surface and be exposed to the environment, forming a cell mimetic membrane structure and reducing the transplant reaction of cell line cells to PCn moieties connected SWCNTs and ultimately improving cell compatibility of SWCNT-PCn. Moreover, the polyelectrolyte properties of PCn chains also changed the covalent-bond surface of SWCNTs, converting the covalent-bond surface to an electrolyte surface, wherein the compatibility of nanotubes with the cell membrane would be improved. Apparently, this conversion would certainly afford improvements in the cell compatibility and reduction in cytotoxicity.

Although the modification on SWCNTs with PC and PCn moieties reduced the cytotoxicity of SWCNTs, it should be noted that the modified SWCNTs still presented cytotoxicity at some level when higher exposure dosages were applied. For HUVEC and RAW264.7 cell lines, the phenomenon was investigated in repeated experiments. The results indicated that the modifications on SWCNTs were not able to completely remove the cytotoxicity of SWCNTs. We speculated that the cytotoxicity of modified SWCNTs at a high dosage was associated with their extremely high aspect ratio. The SWCNTs and modified SWCNTs can probably be embedded into cell membrane and influence its integrity and further disturb cell proliferation. This assumption has been proved in the following experiments to investigate the interaction of cells with carbon nanotubes using fluorescence microscope on a live cell station.

Using the same SWCNTs and modified SWCNTs with same dosage as we used in our experiments described above and under the same incubation conditions, the obtained cytotoxicity showed significant difference for different cell lines. In general, SWCNTs and modified SWCNTs showed the severest cytotoxicity against HUVEC, followed by RAW264.7, while L929 performed the best tolerance for the nanotubes. The difference is certainly related to the different characters of cell lines. HUVEC is a primary cell line coming from human beings, which presented a weak tolerance against hazardous materials such as carbon nanotubes. RAW264.7 and L929 are both murine tumor-cells which have been subcultured for several times, thereby exhibiting higher tolerance to hazardous materials. Moreover, as a macrophage, RAW264.7 tends to uptake while L929 tends to exclude foreign matters; therefore the former one is more susceptible to the effects of hazardous materials. Additionally, the differences of the cell membrane structures, protein expressions, and so forth would inevitably affect the cell proliferation, thereby impacting the cytotoxicity results. More works should be carried out to study the effects of hazardous materials on gene mutation, growth factors, cell receptors, transcription factors, cellular signaling pathways, and so on. The observation that different cell lines show different cytotoxicity results under the same conditions suggests that different conclusions, and sometimes even the opposite opinions, might be drawn based on which cell lines were chosen for nanotoxicological studies. Also, our results indicate that more careful studies on the bioeffects on these nanomaterials are necessary.

### 3.4. Fluorescent Microscopy Investigation at 37°C

**Figure 5:**
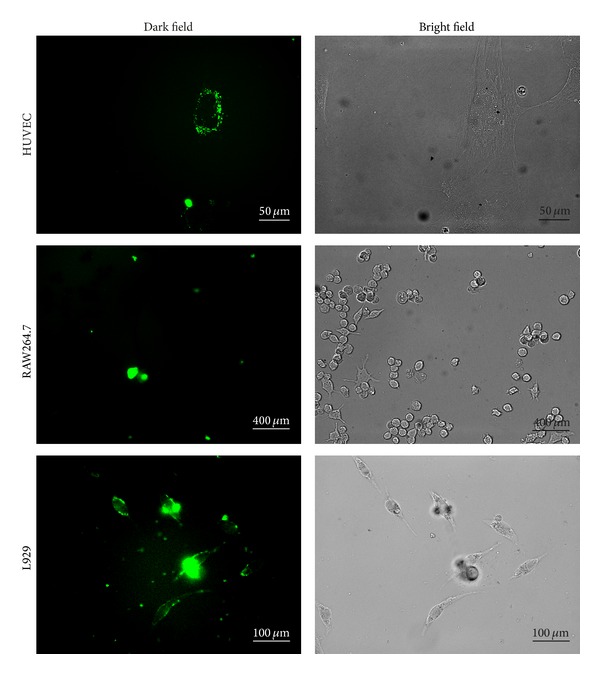
The fluorescence microscopy images of HUVEC, RAW264.7, and L929 incubated with SWCNT-PCn-FITC for 24 h at 37°C with dose of 20 *μ*g/mL.

In order to further investigate the mechanism that causes cytotoxicity of the modified SWCNTs, fluorescent microscopy investigation was used. A fluorophores, FITC, was connected to SWCNT-PCn. Subsequently, a Carl Zeiss Axio Scope A1 fluorescent microscopy equipped with a water immersion/dipping objective lens was utilized to observe the interaction of the various cells and the fluorescently-labeled SWCNT-PCn (SWCNT-PCn-FITC) in a live cell station.  Figure 5 shows the photographs taken 24 hours after incubating the cells with SWCNT-PCn-FITC at 37°C with a dosage of 20 *μ*g/mL.

As shown in Figure 5, it was obvious that SWCNT-PCn-FITC was internalized by cells in RAW264.7, L929, and HUVEC. As macrophage which is highly specialized in removing of dying cells, dead cells and cellular debris, RAW264.7 was also well known for its phagocytosis of ingesting foreign materials [52]. Carbon nanotubes, both pristine CNTs and modified ones, were subjected to uptake by RAW264.7 [28]. Our data of phosphoryl choline modified SWCNTs was in accordance with that phenomenon. However, in HUVEC and L929 cells without phagocytosis, the fluorescence was still strong enough to be regarded as that of SWCNT-PCn-FITC was taken up by the cells. Based on this observation, we assume that the internalization of SWCNT-PCn-FITC was not only related to the phagocytosis but also involved in passive transport. Besides our results of L929 and HUVEC, the passive transport was also documented by other researchers in various cells [33, 53].

Besides the images shown in Figure 5, more data acquired during our observation of the living cells under microscope in live cell station showed that not all cells took up SWCNT-PCn-FITC. Those cells which internalized modified SWCNTs were abnormal cells showing irregular morphology, apoptotic and necrotic, and cell death, while the cells which were not occupied by modified SWCNT appeared to be normal. This difference suggested that the phosphoryl choline modified SWCNT still showed a certain degree of cytotoxicity just as concluded in the CCK-8 assay. In other words, the modification on SWCNTs by phosphoryl choline moieties can significantly reduce the cytotoxicity of SWCNTs but cannot eliminate it completely.

More remarkably, in CCK-8 assay with a dosage of 20 *μ*g/mL, SWCNT-PCn showed severest cytotoxicity against RAW264.7, which is followed by HUVEC, while cell viability of L929 was not significantly different from the control group. The same trend has also been seen in investigation under live cell station: under microscope, RAW264.7 group showed the largest number of cells which took up SWCNT-PCn-FITC. All these findings suggest that the use of only statistical CCK-8 cell viability assay is not enough for evaluating cytotoxicity of SWCNTs-like nanomaterials. Statistical CCK-8 cell viability assay probably only reflected the cell that internalized hazardous materials rather than the overall destructiveness on cells. This thought leads to another explanation of the mechanism of how cytotoxicity was attenuated by phosphoryl choline modifications on SWCNTs: the phosphoryl choline moieties grafted on SWCNTs rendered SWCNTs hydrophility and reduced the ratio of cells which internalized SWCNTs, resulting in a significant decrease in cytotoxicity. Based on that, more deliberate examinations of interactions of nanomaterials with cells, especially with subcellular organelles, should be carried out; since only in this way comprehensive and reliable results of cytotoxicity of these materials would be obtained.

### 3.5. Fluorescent Microscopy Investigation at 4°C

When SWCNT-PCn-FITC was incubated with cells under normal incubation temperature, the internalization by cells could be seen clearly from the above sections. It is well known [54] that the internalization of nanoparticles can be subdivided into two categories: namely, receptor-mediated endocytosis and macropinocytosis. The former is fast and dependent on energy (active transport), while the latter shows no temperature dependence (passive transport). Thus, by incubating cells along with materials at different temperature and investigating the endocytosis of materials in cells, we can understand the subdivided pathway and the cytotoxicity caused by modified SWCNT nanoparticles.

**Figure 6:**
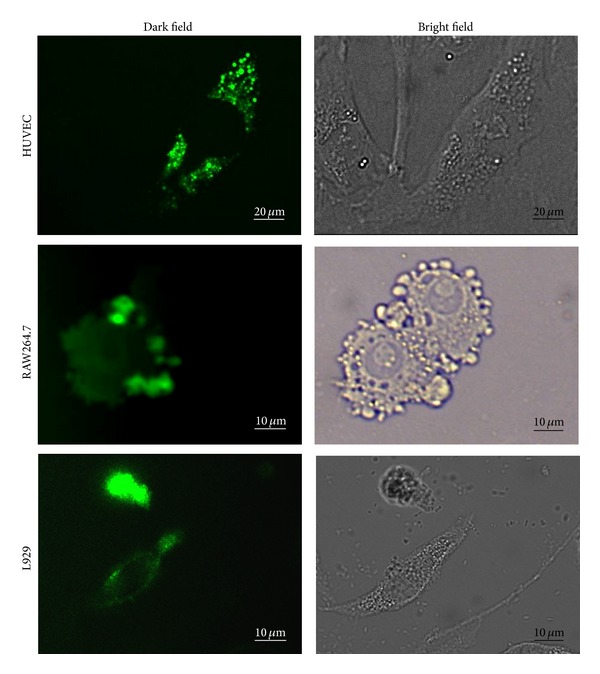
The fluorescence microscopy images of HUVEC, RAW264.7, and L929 incubated with SWCNT-PCn-FITC for 4 h at 4°C with dose of 20 *μ*g/mL.

The cells were incubated with SWCNT-PCn-FITC under lower temperature at 4°C. Unlike active transport, passive transport does not require an input of energy. The rate of passive transport depends on the permeability of the cell membrane, which, in turn, depends on the organization and characteristics of the membrane lipids and proteins [55]. If cells were incubated under low temperature, the active transport would be inhibited while passive transport would remain the same.  Figure 6 shows the fluorescence microscopy images of HUVEC, RAW264.7, and L929 incubating with SWCNT-PCn-FITC for 4 h at 4°C with a 20 *μ*g/mL dose. The fluorescence was still strong enough for the confirmation that SWCNT-PCn-FITC has been taken by all three kinds of cells. This fact illustrated that passive transport contributes to the internalization of modified SWCNT by cells. Furthermore, as shown in the image of RAW264.7, the fluorescence in the cells decreased significantly, implying that the phagocytosis of the cell was inhibited by low-temperature incubation, which in turn led to decrease in cell vitality followed by decreased chemical energy input and ultimately suppression of the active transport process. However, the strong fluorescence on cell membranes as well as the vesicular structures attached on cells still suggested the existence of SWCNT-PCn-FITC, which probably mainly resulted from passive transport. Continuing the observation at 4°C, we found that the cells which contained modified SWCNTs were also in their abnormal states just as what we found under higher temperature, revealing the impacts of nanomaterials on cells and the cytotoxicity nanomaterial would cause.

By comparing the images taken at 37°C and 4°C, we proposed that the internalization of modified SWCNTs in cells was caused by both active and passive transport and also depended on cell line itself. At lower temperature, the passive transport dominated the internalization process and could still cause the damage on cells.

## 4. Conclusions

In conclusion, two kinds of modified SWCNTs were prepared by grafting small molecule phosphoryl choline moiety and polymeric phosphoryl choline chains, respectively. The modified products showed great extent of hydrophilization. Moreover, the modified SWCNTs were found to be able to disperse stably in water and cell culture medium even a long time after incubation, which provided us with an opportunity to evaluate the cytotoxicity of SWCNTs with CCK-8 assay. The results indicated that the modification with biocompatible phosphoryl choline moieties greatly reduced but cannot completely eliminate the cytotoxicity of SWCNTs. Furthermore, the investigation under fluorescence microscope in a live cell station suggested that the internalization of modified SWCNTs was caused by both active transport, phagocytosis, and passive transport. The cell-internalized modified SWCNTs were found to be in their abnormal states, which were probably caused by the impacts of SWCNTs on cell membrane. But the percentage of cells which internalized modified SWCNTs decreased significantly due to the hydrophilization of SWCNTs, which is the observation that explained the improvements in cytotoxicity results in CCK-8 assay. Based on this discovery, we proposed that more deliberately designed experiments to investigate the interactions of nanomaterials with cells, especially to subcellular organelles, need to be carried out, since, only in this way, comprehensive and reliable results of cytotoxicity of these nanomaterials can be obtained.

## Figures and Tables

**Table 1:** Physicochemical characterization of the SWCNTs and modified SWCNTs.

	Pristine SWCNTs	SWCNT-PC	SWCNT-PCn
Diameter (nm)	3–5	3–5	3–5
Length (*μ*m)	5–15	5–15	5–15
Purity (%)	97.52	98.76	98.94
Zeta potential in H_2_O (mV)	−36.7	−12.4	−10.5
Metal impurities (ppm)	Fe 152, Ni 89	Fe 110, Ni 56	Fe 106, Ni 54, Cu 87
